# Gambling Behaviour, Motivations, and Gender Differences Among Medical Students in Poland: Survey-Based Study

**DOI:** 10.3390/healthcare13202555

**Published:** 2025-10-10

**Authors:** Dominik Krupka, Jerzy Brzoza, Olgierd Cugier, Maciej Szwajkowski, Jagoda Szwach, Magdalena Raczkowska, Adam Chełmoński, Julia Drewniowska

**Affiliations:** Student Scientific Club of Transplantology and Advanced Therapies of Heart Failure, Institute of Heart Diseases, Faculty of Medicine, Wroclaw Medical University, 50-368 Wroclaw, Poland

**Keywords:** gambling disorder, pathological gambling, gambling motives, behavioural addictions, medical students, South Oaks Gambling Screen (SOGS), survey-based research, gender differences

## Abstract

**Background:** In psychiatry, gambling is classified as an addiction-related disorder and is characterized by a persistent, problematic pattern of behaviour that leads to significant distress and functional impairment. This study aims to explore the prevalence, underlying motivations, and potential academic impact of gambling behaviours among medical students in Poland. **Methods:** An anonymous online survey was conducted among students from multiple medical universities across Poland. Participants completed a sociodemographic questionnaire and the South Oaks Gambling Screen (SOGS). Respondents who reported any past or current gambling activity were additionally asked about their motivations and potential academic consequences. **Results:** The study included 281 participants. Active or past gambling was reported by 55% of respondents, with men significantly more likely to gamble currently. Women were predominantly non-problem gamblers, whereas men more often scored within the “some problems” range on the SOGS. Motivations also differed: women emphasised financial gain, while men cited fun, socializing, and competition. Lottery and scratch cards were most popular overall, though men preferred skill-based and casino activities. **Conclusions:** Although participants showed relatively low levels of gambling involvement, their risk of developing pathological gambling was comparable to that of the general population. Gender influenced involvement in different gambling patterns.

## 1. Introduction

Gambling is defined as a persistent, problematic pattern of behaviour that leads to clinically significant impairment, disrupts daily functioning, and results in considerable distress. Initially classified as an impulse control disorder, gambling was redefined in the fifth edition of the *Diagnostic and Statistical Manual of Mental Disorders* as an addiction-related disorder. The diagnosis of gambling disorder (GD, plural: GDs) requires meeting at least four of nine diagnostic criteria within a 12-month period [1].

Gambling addiction is currently an escalating issue of global concern [2]. According to the World Health Organization (WHO), the 12-month prevalence of pathological gambling (PG) among adults ranges from 0.1% to 6%, depending on country and methodology [3]. Furthermore, a 2024 meta-analysis of 342 studies across 68 countries reported the highest prevalence rates in North America (20.6% for any-risk gambling; 4.7% for problem gambling) and Eastern Europe (21.9% and 4.0%, respectively) [4].

The preferred type of gambling differs by gender, with men more often engaging in strategic, face-to-face forms, while women tend to choose non-strategic activities such as bingo [5,6]. Online gambling has shown a marked increase in recent years, and it is emerging as a growing health concern among both sexes [7,8].

Healthcare workers are among the professional groups most exposed to stress in their daily practice. This often leads to mental health problems and increases the risk of addictive behaviours, including compulsive gambling [9,10].

The problem of gambling is therefore evident not only among healthcare professionals but also among medical students, who will eventually join the healthcare system [11,12].

Scandroglio et al. assessed gambling among medical students using the Italian version of the South Oaks Gambling Screen (SOGS) and a sociodemographic questionnaire. In the study, 8.7% demonstrated gambling-related problems, while 1.5% exhibited characteristics of PG [13,14]. In a cross-sectional survey of 403 medical and dental students, Aderinto et al. also used the SOGS questionnaire, revealing that 40.2% engaged in gambling activities. Furthermore, 10.4% scored above the threshold for a potential gambling disorder [11].

Unfortunately, education and awareness regarding the negative consequences of gambling, as well as the underlying psychological mechanisms, remain insufficient in these professional groups. This phenomenon contributes to the underestimation of the gambling problem and to the infrequent use of screening in everyday medical practice [15,16]. This situation, further driven by the expanding availability of online gambling platforms, may further increase gambling prevalence and the associated socioeconomic problems [17,18,19].

This study aims to explore the phenomenon of gambling among medical students at Polish universities, with particular attention paid to gender differences in gambling patterns, as well as its underlying causes and consequences.

## 2. Materials and Methods

### 2.1. Participants and Procedure

This study was conducted in the first half of August 2025, during the 2024/2025 Academic Year. Survey invitations were distributed via social media channels connected with academic medical organizations and medical school societies from 20 universities located in Wrocław, Katowice, Poznań, Łódź, Warsaw, Lublin, Kraków, Bydgoszcz, Rzeszów, Zielona Góra, Opole, Szczecin, Gdańsk, Białystok, Olsztyn, Kielce, Czestochowa, and Płock. The utilised platforms involved Messenger, Facebook, and Instagram.

Participation in the survey was voluntary and fully anonymous. The only eligibility criterion was current enrolment as a medical student at a Polish medical university; no exclusion criteria were applied. Respondents who consented were requested to complete a sociodemographic questionnaire and the Polish, validated adaptation of the SOGS [14].

### 2.2. Measures

#### 2.2.1. Sociodemographic Characteristics

The participants were asked to provide information, including their age, sex, the name of their medical university, year of study, hometown population size, type of residence during the semester, main source of income, employment status while studying, academic difficulties, relationship status, educational level, and family history of mental health problems. At the end of the first section, respondents were asked whether they had ever engaged in, or were currently engaging in, at least one of the listed types of gambling or any other form of gambling.

If a respondent indicated current or past involvement in any form of gambling in the final question of the first section, the questionnaire proceeded to the second section, which was inspired by the Reasons for Gambling Questionnaire (RGQ) and adapted for our study [20].

This section covered gambling motivations, preferred mode of gambling (land-based or online), and the occurrence of any academic problems resulting from gambling, which included absences from lessons, insufficient preparation for examinations, deterioration of social relationships with peers, and other factors.

#### 2.2.2. South Oaks Gambling Screen

The SOGS questionnaire is one of the most widely used tools for assessing the prevalence of PG in a given population [13,21].

The Polish version of the SOGS was validated by Wieczorek et al. to effectively assess the severity of gambling problems in the Polish population. This process involved translation, cultural adaptation, and psychometric evaluation of reliability. The Polish adaptation differs from the original. In the Polish version, the most optimal cut-off score for identifying individuals who should undergo more detailed assessment due to meeting the criteria for PG was set at 7 points, rather than 5 as indicated in the original instructions [22]. However, Wieczorek et al. inconsistently treat 5 points as a cut-off for further PG diagnostics in their final guidelines. Therefore, the value of 5 points was applied in the present study to stay consistent with international research.

The questionnaire contains 16 items, of which 12 contribute to the final score. The remaining items serve to identify the type of gambling, daily spending, and the presence of relatives or acquaintances with gambling problems. The first item addresses the gambling frequency of specific behaviours, while the others use a “yes/no” format. The method used to calculate the final score is presented in Table 1.

### 2.3. Data Analysis

The study population was stratified into two groups: individuals with current or past gambling experience (gambling group [GG]) and those who had never engaged in gambling (non-gambling group [non-GG]). This classification was applied in the overall cohort characterization. For the analysis of gambling prevalence, SOGS scores, and gambling-related motivations, participants were further stratified by sex (female vs. male).

The Shapiro–Wilk test was used to assess the normality of distributions for each subgroup. Non-normally distributed data were presented as medians with interquartile ranges. For comparisons involving non-normally distributed variables, the Mann–Whitney U test was applied. Categorical variables were presented as frequencies and percentages, and differences between groups were assessed using Pearson’s chi-square test. A *p*-value of <0.05 was considered statistically significant.

Data analysis was performed using STATISTICA 13.3 software, licensed to Wrocław Medical University.

## 3. Results

### 3.1. General Characteristics

The study included 281 participants with a median age of 22 (21–24) years, 221 (80%) female. Median age was higher in the GG, at 23 years (21–24), compared to 22 years (21–24) in the non-GG, *p* = 0.04. The proportion of women was lower in the GG (72% vs. 88%, *p* = 0.001). Residency during the semester also differed, with the GG most frequently living in a shared rented apartment (25%) or at a family/relative’s home (25%) compared to 20% and 23%, respectively, in the non-GG, *p* = 0.02. Additionally, individuals in the GG were more likely to be in a relationship (62% vs. 52% in the non-gambling group), *p* = 0.02. Statistically significant values are shown in Table 2.

### 3.2. Prevalence of Gambling Activity

Most respondents reported either current (17%) or past (38%) gambling. Females were more likely to report never gambling (50%) or past gambling (38%), while males reported current gambling more often (37%), *p* < 0.001. Detailed characteristics are presented in Table 3.

### 3.3. Forms of Gambling Participation

Among participants with present or past gambling experience, SOGS scores differed by gender: more women were present in the ‘non-problem gambling’ (0 points) group (80%) and more men fell in the ‘some problems with gambling’ (1–4 points) group (40%). The prevalence of ‘probable pathological gambling’ (≥5 points) was consistent among the groups at 2%, *p* = 0.014. Median SOGS score was 0 (0–0) in women and 0 (0–2) in men, with a significant difference between groups, *p* = 0.002. Notably, all participants (100%) denied experiencing any gambling-related problems with studying medicine, including missed classes, poor exam preparation, social difficulties, and other issues.

A significant majority of respondents reported gambling exclusively in land-based settings (67% of females, 43% of males), whereas men were more likely than women to engage in both online and offline gambling activities (41% vs. 22%), *p* = 0.03.

Men were more inclined than women to outline the following reasons for gambling: excitement or fun (60% vs. 26%), *p* < 0.001, as a free time activity (43% vs. 13%), *p* < 0.001, to escape boredom or fill the time (29% vs. 6%), *p* = 0.002, sense of competition or to impress others (21% vs. 5%), *p* = 0.01, and socializing with friends or relatives (62% vs. 26%), *p* < 0.001. Females more than men frequently cited earning money or winning a grand prize as their motivation (55% vs. 31%), *p* = 0.03. No significant gender differences were observed for satisfaction when winning, gambling to relax, or fear of missing out on a win. Table 4 presents the full characteristics.

### 3.4. South Oaks Gambling Screen Results

Numerical lotteries (72%), scratch cards (70%), playing cards (32%), and sports betting (30%) were the most popular gambling activities in the population. Gambling preferences differed by gender: men were more likely than women to play card games (59% vs. 21%), *p* < 0.001, betting on animals (17% vs. 7%), *p* = 0.03, going to a casino (29% vs. 3%), *p* < 0.001, trading at the stock market (29% vs. 3%), *p* < 0.001, playing skill games for money (26% vs. 7%), *p* = 0.007, and engaging in other forms of gambling (9% vs. 1%), *p* = 0.03. On the contrary, women tend to play scratch cards and other ‘paper’ games more often than men (78% vs. 50%), *p* = 0.005, or to participate in number games and lotteries (79% vs. 55%), *p* = 0.007.

Respondents declared various daily gambling expenditures, but most fell within a 5 Polish zloty (PLN) to 500 PLN range. Men generally gambled larger amounts, with 19% stating risking more than 500 PLN on one day, *p* < 0.001. In contrast, two women reported gambling over 5000 PLN on one day.

Respondents rarely outlined any individuals in their environment with a past or present gambling problem (*n* = 30, 20%). The most prevalent answers were: ‘other relative’ (*n* = 14), ‘friend or other important person’ (*n* = 9), and father (*n* = 6).

Most gamblers never attempt to win back the money they lost (88%), with no statistical difference between genders. Similarly, most never denied losing money (95%). Moreover, the vast majority of responders did not perceive their gambling or money betting as a problem (97%).

Men more frequently gambled more than they intended (33% vs. 12% in women), *p* = 0.002. Moreover, the male population is at greater risk of losing time from important activities such as job or school due to their addiction (12% and 1%, respectively), *p* = 0.008.

Responses to other questions provide no distinctions between the genders. Most respondents stated not feeling criticised for gambling (97%), not feeling guilty about gambling (93%), not feeling unable to stop gambling (97%), or not hiding any evidence of gambling activities (95%). Almost all respondents (99%) denied failing to repay money borrowed for gambling. Overall, they rarely borrowed money to gamble, with the most common stated sources being household money (5%), relatives and in-laws (1%), and selling property (1%).

Statistically significant sex-stratified SOGS results are presented in Table 5. Full versions of each Table can be found in the Supplementary Materials.

## 4. Discussion

In 2024, the Polish Public Opinion Research Center (PPORC) published a report on gambling and other behavioural addictions in Poland, which showed that 31.7% of Poles had gambled for money at least once in the previous year and 1.9% of gamblers (0.6% of the general population) were at risk of possible gambling addiction. These results suggest that this is a common phenomenon in Poland [23].

In our study, 55% of respondents had ever (currently or in the past) participated in gambling activities. Among them, 24% belong to the group that may potentially have gambling-related problems (SOGS score between 1 and 4), and 2% are considered probable pathological gamblers (SOGS score ≥ 5). Comparable results were found in the studies, which also used the SOGS questionnaire, made by Scandroglio et al. (2022) [4], who identified 1.43% probable PG among healthcare students, and by Azevedo et al. (2023) [14], who reported 3.1% among students in Porto. Prevalence of GDs among adults reported by the WHO ranges from 0.1% to 5.8% [24], and a similar pattern can be observed in Azevedo et al. study [25]. Importantly, the estimated prevalence of possible GD in our sample was 0.7% (3 respondents with SOGS score ≥ 5 out of 281 participants), a result that corresponds with population-level estimates [23], which supports the reliability of our findings. This may be explained by the fact that although medical students are at risk of developing GDs due to stress, chronic fatigue, or academic pressure, they may be more aware of addiction mechanisms and thus self-protect better. This might have arisen from, presumably, parents’ higher education (81% of responders) or practical medical knowledge they acquired during their studies. However, these are only speculative assumptions that require further investigation.

GDs are more prevalent in men than in women and occur more frequently among young and middle-aged individuals compared to older adults [1]. Gambling preferences differ by gender. Men tend to choose skill-based and strategic forms of gambling, while women more commonly engage in luck-based activities [5,6]. Our analysis revealed that scratch cards were a more present form of gambling in a female subpopulation compared to males, which remains in accordance with Scandroglio et al. study results [13]. Playing cards was the most popular gambling activity among men, which is consistent with their higher tendency to report socialising and competition as motivations for gambling, compared to women.

Our study aimed to assess the prevalence of gambling behaviours among medical students, as they represent an at-risk group due to high academic pressure, chronic stress exposure, and emotional fatigue. Research shows a common co-occurrence of GDs and a range of psychiatric conditions, including depressive and bipolar disorders, antisocial personality disorder, and substance use disorders, particularly alcohol use disorder [26,27,28]. Moreover, a range of psychological and temporal factors, such as boredom susceptibility, peer influence, and self-destructive tendencies, contribute to adolescent gambling behaviours. Furthermore, cognitive distortions, sensation-seeking, and temporal perspectives have been consistently associated with increased gambling severity [29,30].

The likelihood of engaging in gambling also increases significantly in the context of severe stress [31]. In the psychology of gambling addiction, particular attention has been given to the concept of “escape motivation.” This refers to gambling undertaken as a means of avoiding or alleviating negative emotional states such as stress, anxiety, or loneliness. In this context, the primary drive is not the prospect of monetary gain but rather the regulation of mood [32,33]. Interestingly, the results of our publication indicate otherwise, winning money and excitement were the most common motivations for both genders. Our results align with findings by López-del-Hoyo et al., who observed a similar trend in their study conducted in 2022 [34].

The results indicate that among students in the GG, the majority were in a relationship (formal or informal). Grant et al. study [35] shows a similar pattern but not statistically significant. However, there is a deficiency of research examining the correlation between relationship status and gambling behaviour among students. The above underscores the necessity for further comprehensive research.

Male gender is a well-known risk factor for developing gambling addiction, which would confirm our outcomes that males are more often found in the at-risk group (SOGS score 1–4) than women. These results are consistent with studies of Scandroglio et al. [13] (27.45% in men vs. 4.48% in women, *p* < 0.001) and Giralt et al. [36] (6.4% in males vs. 1.5% in females, *p* = 0.001). Greater male engagement in gambling activities (37% vs. 11% in women) may indicate more continuous involvement, which is widely documented in the literature [37,38]. Our study also found that men more frequently gambled for longer than they intended. Moreover, a higher proportion of male students reported losing time from essential activities such as work or school due to gambling, which is consistent with previous data. [4,39]. The above phenomenon reveals a pattern of heightened male vulnerability to problem gambling. Mancini et al., in their study, attribute this vulnerability to specific developmental and psychosocial factors [39].

It is important to highlight the limitations of our research. Firstly, the study was limited by a relatively small sample size, as only 281 students chose to participate. This may be due to limited access to information about the study, as it was distributed solely through social media rather than via the official university communication channels, such as the institutional e-mail system. It is necessary for further studies to investigate this problem with a larger sample size to reduce sampling errors and increase the population’s representation. Secondly, in our sample, females were the majority (80%, *n* = 221) and therefore the findings may be influenced by this gender distribution. Moreover, our study is not randomised, as only volunteers completed the questionnaire. Due to the sensitive nature of the topic—gambling addiction—some students may have chosen not to participate or left the questionnaire before completion. Additionally, the possibility of underreporting due to social desirability bias should be acknowledged, because all participants stated that they had not encountered any gambling-related problems affecting their medical studies, such as skipping classes, neglecting exam preparation, or facing other issues. This concern is especially pertinent in the case of medical students, who may experience a strong inclination to portray themselves in a responsible and socially acceptable manner [40]. Finally, it is important to note that our study did not address psychometric measures such as anxiety, stress, or impulsivity, which limits the ability to capture important psychological factors associated with gambling behaviour.

## 5. Conclusions

Issues addressed in our study are primarily focused on the differences in approach to gambling in both genders. We considered sociodemographic characteristics, motivation, forms, methods, and possible addiction risk of gambling. We found the SOGS questionnaire useful in enabling comparisons of results between different research institutions. Survey data indicate clear gender differences in gambling patterns—a factor that should be incorporated into future research and preventive strategies. The prevalence rate of possible PG identified in our study aligns with that reported by PPORC. This finding is particularly significant, as it is representative of the general population; therefore, it is important to further expand research in this area. These findings point to the need for more comprehensive training of medical students in recognizing gambling-related problems and their consequences, both for personal prevention and future patient care. Additionally, incorporating gambling-related awareness into medical curricula could enhance future prevention and intervention efforts.

## Figures and Tables

**Table 1:** Scoring system of the South Oaks Gambling Screen (SOGS).

Question(s)	Scoring Criteria	Maximum Points
Q4	1 point if answered *“most of the time I lose”* or *“every time I lose”*	1
Q5	1 point if answered *“yes, less than half of the times I’ve lost”* or *“yes, most of the time”*	1
Q6	1 point if answered *“yes, in the past, but not now”* or *“yes”*	1
Q7–Q11	1 point for each single *“yes”*	5
Q13–Q16 (except 16j and 16k)	1 point for each single *“yes”*	6
**Total**		**0–20**

**Table 2:** General characteristics of the study population.

Parameter		All Participants (*n* = 281)	Valid *n*	Group: Gambling Count (*n* = 153)	Group: Non-Gambling Count (*n* = 128)	*p*-Value
Age (IQR)		22 (21–24)	281	23 (21–24)	22 (21–24)	0.04
Sex, female (%)		221 (80)	278	110 (72)	111 (88)	0.001
Place of residence during the semester, *n* (%)	Rented apartment shared with another person	65 (23)	281	39 (25)	26 (20)	0.02
	Rented an apartment with a partner	48 (17)		28 (18)	20 (16)	
	Rented an apartment alone	61 (22)		36 (24)	25 (20)	
	Rented a room/lodging	26 (9)		9 (6)	17 (13)	
	Family/relative’s home	68 (24)		39 (25)	29 (23)	
	Student dormitory	13 (5)		2 (1)	11 (9)	
Relationship, *n* (%)	No	118 (42)	278	57 (38)	61 (48)	0.02
	Informal	142 (51)		79 (52)	63 (50)	
	Formal	18 (6)		15 (10)	3 (2)	

Other sources of income—partner’s financial support, begging, student loans or other loans, cryptocurrencies, need-based scholarship, and parental support, work and parental support, sports betting, and others; Other forms of employment—planning to start work in the upcoming academic year, previously employed, private practice with sole proprietorship, work very rarely.

**Table 3:** Prevalence of gambling activities among participants.

Parameter		All Participants (*n* = 281)	Valid *n*	Group: Female (*n* = 221)	Group: Male (*n* = 57)	*p*-Value
Ever taking part in gambling activities such as listed below ^1^, *n* (%)	Never	126 (45)		111 (50)	15 (26)	
	Yes, in the past	106 (38)	278	85 (38)	21 (37)	<0.001
	Yes, at the moment	46 (17)		25 (11)	21 (37)	

^1^ Playing cards or dice games for money, betting on animals or sports, going to a casino (legal or otherwise), playing a lottery, buying scratch cards, playing slot machines or other gambling machines, playing on the stock market, playing games of skill such as bowling for money, and other non-specified gambling activities.

**Table 4:** Patterns and forms of gambling participation in the study population.

Parameter		Gambling Participants (*n* = 152)	Valid *n*	Group: Female (*n* = 110)	Group: Male (*n* = 42)	*p*-Value
SOGS score, *n* (%)	0 points = non-problem gambling	112 (74)		88 (80)	24 (57)	
	1–4 points = some problems with gambling	37 (24)	152	20 (18)	17 (40)	0.014
	≥5 points = probable pathological gambling	3 (2)		2 (2)	1 (2)	
SOGS score (IQR)		0 (0–1)	152	0 (0)	0(0–2)	0.002
The type of gambling method, *n* (%)	Only online	20 (13)		13 (12)	7 (17)	0.03
	Online and land-based	41 (27)	152	24 (22)	17 (41)	
	Only land-based	91 (60)		73 (67)	18 (43)	
Reasons for gambling, *n* (%)	A. Excitement or fun, yes	54 (36)	152	29 (26)	25 (60)	<0.001
	B. Satisfaction when winning, yes	50 (33)		31 (28)	19 (45)	0.13
	C. Hobby or free time activity, yes	32 (21)		14 (13)	18 (43)	<0.001
	D. To fill the time or escape boredom, yes	19 (13)		7 (6)	12 (29)	0.002
	E. To relax, yes	22 (14)		14 (13)	8 (19)	0.63
	F. To compete or to impress others, yes	14 (9)		5 (5)	9 (21)	0.01
	G. *“I won’t win if I don’t play”*, yes	5 (3)		2 (2)	3 (7)	0.31
	H. As a social activity, yes	55 (36)		29 (26)	26 (62)	<0.001
	I. To earn money or to win big money, yes	73 (48)		60 (55)	13 (31)	<0.03

**Table 5:** Comparison of South Oaks Gambling Screen results between male and female respondents.

SOGS Question		Gambling Participants (*n* = 152)	Valid *n*	Group: Female (*n* = 110)	Group: Male (*n* = 42)	*p*-Value
**1. Indicate which of the following types of** **gambling you have** **done** **in your lifetime.**						
Played cards for money, *n* (%)	not at all	104 (68)		87 (79)	17 (40)	
	less than once a week	46 (30)	152	22 (20)	24 (57)	<0.001
	once a week or more	2 (1)		1 (1)	1 (2)	
Bet on horses, dogs or other animals, *n* (%)	not at all	138 (91)		103 (94)	35 (83)	
	less than once a week	12 (8)	152	5 (5)	7 (17)	0.03
	once a week or more	2 (1)		2 (2)	0 (0)	
Went to casino (legal or otherwise), *n* (%)	not at all	137 (90)		107 (97)	30 (71)	
	less than once a week	14 (9)	152	2 (2)	12 (29)	<0.001
	once a week or more	1 (1)		1 (1)	0 (0)	
Played the numbers or bet on lotteries, *n* (%)	not at all	43 (28)		24 (22)	19 (45)	
	less than once a week	102 (67)	152	82 (75)	20 (48)	0.007
	once a week or more	7 (5)		4 (4)	3 (7)	
Played the stock and/or commodities market, *n* (%)	not at all	137 (90)		107 (97)	30 (71)	
	less than once a week	7 (5)	152	2 (2)	5 (12)	<0.001
	once a week or more	8 (5)		1 (1)	7 (17)	
Bowled, shot pool, played golf or played some other game of skill for money, *n* (%)	not at all	133 (88)		102 (93)	31 (74)	
	less than once a week	17 (11)	152	7 (6)	10 (24)	0.007
	once a week or more	2 (1)		1 (1)	1 (2)	
Pull tabs or “paper” games other than lotteries, *n* (%)	not at all	46 (30)		25 (23)	21 (50)	
	less than once a week	102 (67)	152	82 (75)	20 (48)	0.005
	once a week or more	4 (3)		3 (3)	1 (2)	
Engage in some form of gambling not listed above, *n* (%)	not at all	147 (97)		109 (99)	38 (90)	
	less than once a week	4 (3)	152	1 (1)	3 (7)	0.03
	once a week or more	1 (1)		0 (0)	1 (2)	
**2. What is the largest amount of money you have ever gambled with on any one day?**						
I have never gambled, *n* (%)		4 (3)	152	4 (4)	0 (0)	<0.001
5 PLN ^1^ or less, *n* (%)		37 (24)		34 (31)	3 (7)	
More than 5 PLN but less than 50 PLN, *n* (%)		73 (48)		57 (52)	16 (38)	
More than 50 PLN but less than 500 PLN, *n* (%)		27 (18)		12 (11)	15 (36)	
More than 500 PLN but less than 5000 PLN, *n* (%)		9 (6)		1 (1)	8 (19)	
More than 5000 PLN but less than 50,000 PLN, *n* (%)		2 (1)		2 (2)	0 (0)	
More than 50,000 PLN, *n* (%)		0 (0)		0 (0)	0 (0)	
**7. Did you ever gamble more** **than you intended to?**, yes, *n* (%)		27 (18)	152	13 (12)	14 (33)	0.002
**15. Have you ever lost time from work (or school) due to betting or gambling?,** yes, *n* (%)		6 (4)	152	1 (1)	5 (12)	0.008

^1^ Polish Zloty.

## Data Availability

Data are contained within the article.

## Supplementary Materials

The following supporting information can be downloaded at: https://www.mdpi.com/article/10.3390/healthcare13202555/s1, Table S1. Full version of Table 2. General characteristics of the study population. Table S2. Full version of Table 3. Prevalence of gambling activities among participants. Table S3. Full version of Table 4. Patterns and forms of gambling participation in the study population. Table S4. Full version of Table 5. Comparison of South Oaks Gambling Screen results between male and female respondents.

## Abbreviations

The following abbreviations are used in this manuscript:

GD, plural: GDs	Gambling disorder
WHO	World Health Organization
PG	Pathological gambling
SOGS	South Oaks Gambling Screen
GG	Gambling group
Non-GG	non-gambling group
PLN	Polish zloty
PPORC	Polish Public Opinion Research Center
