# Supplementary material for: Gambling Behaviour, Motivations, and Gender Differences Among Medical Students in Poland: Survey-Based Study
Source: Healthcare (Basel). 2025 Oct 10;13(20):2555. doi: 10.3390/healthcare13202555 (PMC12562915; doi:10.3390/healthcare13202555)
Supplement: Supplementary file 1 [file healthcare-13-02555-s001.zip › healthcare-3865862-supplementary.pdf]

Supplementary Material

Gambling Behaviour, Motivations, and Gender Differences Among Medical Students in Poland: Survey-Based Study

Dominik Krupka †, Jerzy Brzoza *,†, Olgierd Cugier, Maciej Szwajkowski, Jagoda Szwach, Magdalena Raczkowska, Adam Chełmoński and Julia Drewniowska

Student Scientific Club of Transplantology and Advanced Therapies of Heart Failure, Institute of Heart Diseases, Faculty of Medicine, Wroclaw Medical University, 50-368 Wroclaw, Poland

* Correspondence: jerzy.brzoza@student.umw.edu.pl

† These authors (D.K. and J.B.) contributed equally and share equal first authorship.

Table 2. General characteristics of the study population.

Parameter		All participants (n=281)	Valid n	Group: Gambling Count (n=153)	Group: non-Gambling Count (n=128)	p-Value	χ2 (df)
Age (IQR)		22 (21-24)	281	23 (21-24)	22 (21-24)	0.04	-
Sex, female (%)		221 (80)	278	110 (72)	111 (88)	0.001	10.5 (1)
Year of study, n (%)	1	43 (15)	281	24 (16)	19 (15)	0.44	4.8 (5)
	2	64 (23)		31 (20)	33 (26)		
	3	49 (17)		26 (20)	23 (15)		
	4	47 (17)		28 (18)	19 (15)		
	5	55 (20)		31 (20)	24 (19)		
	6	23 (8)		16 (11)	7 (5)		
Hometown, population (%)	Cottage	54 (19)	281	24 (16)	30 (23)	0.32	3.5 (3)
	City < 10^4 people	17 (6)		9 (6)	8 (6)		
	City 10^4 – 10^5 people	68 (24)		36 (24)	32 (25)		
	City > 10^5 people	142 (51)		84 (55)	58 (45)		
Place of residence during the semester, n (%)	Rented apartment shared with another person	65 (23)	281	39 (25)	26 (20)	0.02	14 (5)
	Rented an apartment with a partner	48 (17)		28 (18)	20 (16)		
	Rented an apartment alone	61 (22)		36 (24)	25 (20)		
	Rented a room / lodging	26 (9)		9 (6)	17 (13)		
	Family/relative's home	68 (24)		39 (25)	29 (23)		

	Student dormitory	13 (5)		2 (1)	11 (9)		
Main sources of income, *n* (%)	Scholarships (other than need-based), grants and similar sources	5 (2)	281	2 (1)	3 (2)	0.69	8.2 (11)
	Financial support from parents / other family members	216 (77)		115 (75)	101 (79)		
	Regular or part-time/occasional job	42 (15)		24 (16)	18 (14)		
	Social benefits/welfare support	10 (4)		6 (4)	4 (3)		
	Other sources of income[1]	8 (3)		6 (4)	2 (2)		
Employment during studies, *n* (%)	Never	118 (42)	280	59 (39)	59 (46)	0.42	7.1 (7)
	Occasionally	104 (37)		63 (41)	41 (32)		
	Part-time job	48 (17)		25 (16)	23 (18)		
	Full-time job	6 (2)		4 (3)	2 (2)		
	Other forms of employment[2]	4 (1)		2 (1)	2 (2)		
Learning difficulties, *n* (%)	No	200 (71)	281	113 (74)	87 (68)	0.53	1.3 (2)
	Yes	29 (10)		15 (10)	14 (11)		
	Difficult to say	52 (19)		25 (16)	27 (21)		
Relationship, *n* (%)	No	118 (42)	278	57 (38)	61 (48)	0.02	8 (2)
	Unformal	142 (51)		79 (52)	63 (50)		
	Formal	18 (6)		15 (10)	3 (2)		
Highest parental education, *n* (%)	Primary	3 (1)	281	2 (1)	1 (1)	0.14	5.4 (3)
	Vocational/technical	33 (12)		22 (14)	11 (9)		
	High school	18 (6)		6 (4)	12 (9)		
	University degree	227 (81)		123 (80)	104 (81)		
Mental disorders in family, *n* (%)		115 (47)	244	62 (47)	53 (47)	0.95	0 (1)

[1] Other sources of income - partner's financial support, begging, student loans or other loans, cryptocurrencies, need-based scholarship and parental support, work and parental support, sports betting and others; [2] Other forms of employment - planning to start work in the upcoming academic year, previously employed, private practice with sole proprietorship, work very rarely.

Table 3. Prevalence of gambling activities among participants.

Parameter		All participants (n=281)	Valid n	Group: female (n=221)	Group: male (n=57)	p-Value	χ2 (df)
Ever taking part in gambling activities such as listed below[1], n (%)	Never	126 (45)	278	111 (50)	15 (26)	<0.001	23.6 (2)
	Yes, in the past	106 (38)		85 (38)	21 (37)		
	Yes, at the moment	46 (17)		25 (11)	21 (37)		

[1] Playing cards or dice games for money, betting on animals or sports, going to a casino (legal or otherwise), playing a lottery, buying scratch cards, playing slot machines or other gambling machines, playing on the stock market, playing games of skill such as bowling for money, and other non-specified gambling activities.

Table 4. Patterns and forms of gambling participation in the study population.

Parameter		Gambling participants (n=152)	Valid n	Group: female (n=110)	Group: male (n=42)	p-Value	χ2 (df)
SOGS score, n (%)	0 points = non-problem gambling	112 (74)	152	88 (80)	24 (57)	0.014	8.4 (2)
	1-4 points = some problems with gambling	37 (24)		20 (18)	17 (40)		
	≥5 points = probable pathological gambling	3 (2)		2 (2)	1 (2)		
SOGS score (IQR)		0 (0-1)	152	0 (0)	0(0-2)	0.002	-
The type of gambling method, n (%)	Only online	20 (13)	152	13 (12)	7 (17)	0.03	7.3 (2)
	Online and land-based	41 (27)		24 (22)	17 (41)		
	Only land-based	91 (60)		73 (67)	18 (43)		
Reasons for gambling, n (%)	A. Excitement or fun, yes	54 (36)	152	29 (26)	25 (60)	<0.001	14.6 (1)
	B. Satisfaction when winning, yes	50 (33)		31 (28)	19 (45)	0.13	4.0 (1)
	C. Hobby or free time activity, yes	32 (21)		14 (13)	18 (43)	<0.001	16.6 (1)
	D. To fill the time or escape boredom, yes	19 (13)		7 (6)	12 (29)	0.002	12.2 (1)
	E. To relax, yes	22 (14)		14 (13)	8 (19)	0.63	0.93 (1)
	F. To compete or to impress others, yes	14 (9)		5 (5)	9 (21)	0.01	9.1 (1)
	G. *"I won't win if I don't play"*, yes	5 (3)		2 (2)	3 (7)	0.31	2.4 (1)
	H. As a social activity, yes	55 (36)		29 (26)	26 (62)	<0.001	16.6 (1)
	I. To earn money or to win big money, yes	73 (48)		60 (55)	13 (31)	0.03	6.8 (1)

Table 5. Comparison of South Oaks Gambling Screen results between male and female respondents.

SOGS question		Gambling participants (*n*=152)	Valid *n*	Group: female (*n*=110)	Group: male (*n*=42)	*p*-Value	χ2 (df)
**1. Indicate which of the following types of gambling you have done in your lifetime.**							
Played cards for money, *n* (%)	not at all	104 (68)	152	87 (79)	17 (40)	<0.001	21.0 (2)
	less than once a week	46 (30)		22 (20)	24 (57)		
	once a week or more	2 (1)		1 (1)	1 (2)		
Bet on horses, dogs or other animals, *n* (%)	not at all	138 (91)	152	103 (94)	35 (83)	0.03	6.8 (2)
	less than once a week	12 (8)		5 (5)	7 (17)		
	once a week or more	2 (1)		2 (2)	0 (0)		
Bet on sports, *n* (%)	not at all	106 (70)	152	81 (74)	25 (60)	0.24	2.9 (2)
	less than once a week	33 (22)		21 (19)	12 (29)		
	once a week or more	13 (9)		8 (7)	5 (12)		
Played dice games for money, *n* (%)	not at all	142 (93)	152	101 (92)	41 (98)	0.42	1.7 (2)
	less than once a week	9 (6)		8 (7)	1 (2)		
	once a week or more	1 (1)		1 (1)	0 (0)		
Went to casino (legal or otherwise), *n* (%)	not at all	137 (90)	152	107 (97)	30 (71)	<0.001	26.3 (2)
	less than once a week	14 (9)		2 (2)	12 (29)		
	once a week or more	1 (1)		1 (1)	0 (0)		
Played the numbers or bet on lotteries, *n* (%)	not at all	43 (28)	152	24 (22)	19 (45)	0.007	10.0 (2)
	less than once a week	102 (67)		82 (75)	20 (48)		
	once a week or more	7 (5)		4 (4)	3 (7)		
Played Bingo, *n* (%)	not at all	136 (89)	152	97 (88)	39 (93)	0.64	0.9 (2)
	less than once a week	15 (10)		12 (11)	3 (7)		
	once a week or more	1 (1)		1 (1)	0 (0)		
Played the stock and/or commodities market, *n* (%)	not at all	137 (90)	152	107 (97)	30 (71)	<0.001	23.3 (2)
	less than once a week	7 (5)		2 (2)	5 (12)		
	once a week or more	8 (5)		1 (1)	7 (17)		
Played slot machines, poker machines, or other gambling machines, *n* (%)	not at all	120 (79)	152	89 (81)	31 (74)	0.57	1.1 (2)
	less than once a week	28 (18)		18 (16)	10 (24)		
	once a week or more	4 (3)		3 (3)	1 (2)		
Bowled, shot pool, played golf or played some other game of skill for money, *n* (%)	not at all	133 (88)	152	102 (93)	31 (74)	0.007	10.0 (2)
	less than once a week	17 (11)		7 (6)	10 (24)		
	once a week or more	2 (1)		1 (1)	1 (2)		
Pull tabs or "paper" games other than lotteries, *n* (%)	not at all	46 (30)	152	25 (23)	21 (50)	0.005	10.8 (2)
	less than once a week	102 (67)		82 (75)	20 (48)		
	once a week or more	4 (3)		3 (3)	1 (2)		
Engage in some form of gambling not listed above, *n* (%)	not at all	147 (97)	152	109 (99)	38 (90)	0.03	7.3 (2)
	less than once a week	4 (3)		1 (1)	3 (7)		
	once a week or more	1 (1)		0 (0)	1 (2)		
**2. What is the largest amount of money you have ever gambled with on any one day?**							
I have never gambled, *n* (%)		4 (3)	152	4 (4)	0 (0)	<0.001	38.0 (5)
5 PLN[1] or less, *n* (%)		37 (24)		34 (31)	3 (7)		
More than 5 PLN but less than 50 PLN, *n* (%)		73 (48)		57 (52)	16 (38)		
More than 50 PLN but less than 500 PLN, *n* (%)		27 (18)		12 (11)	15 (36)		

More than 500 PLN but less than 5 000 PLN, *n* (%)	9 (6)		1 (1)	8 (19)		
More than 5 000 PLN but less than 50 000 PLN, *n* (%)	2 (1)		2 (2)	0 (0)		
More than 50 000 PLN, *n* (%)	0 (0)		0 (0)	0 (0)		
**4. When you gamble, how often do you return to win back the money you lost?**						
Never, *n* (%)	134 (88)		99 (90)	35 (83)		
Some of the time (less than half of the times I lost), *n* (%)	13 (9)	152	7 (6)	6 (14)	0.43	2.8 (3)
Most of the times I lost, *n* (%)	4 (3)		3 (3)	1 (2)		
Every time I lost, *n* (%)	1 (1)		1 (1)	0 (0)		
**5. Have you ever claimed to be winning money while gambling, even though you were actually losing money?**						
Never, *n* (%)	145 (95)		107 (97)	38 (90)		
Yes, less than half of the time I lost, *n* (%)	5 (3)	152	2 (2)	3 (7)	0.20	3.3 (2)
Yes, most of the time, *n* (%)	2 (1)		1 (1)	1 (2)		
**6. Do you feel like you have ever had a problem with betting money or gambling?**						
No, *n* (%)	147 (97)		107 (97)	40 (95)		
Yes, in the past, but not now, *n* (%)	1 (1)	152	1 (1)	0 (0)	0.50	1.4 (2)
Yes, *n* (%)	4 (3)		2 (2)	2 (5)		
**7. Did you ever gamble more than you intended to?**, yes, *n* (%)	27 (18)	152	13 (12)	14 (33)	0.002	9.6 (1)
**8. Have people criticised your betting or told you that you had a gambling problem, regardless of whether or not you thought it was true?, yes** , *n* (%)	4 (3)	152	2 (2)	2 (5)	0.65	0.2 (1)
**9. Have you ever felt guilty about the way you gamble or what happens when you gamble?**, yes, *n* (%)	11 (7)	152	6 (5)	5 (12)	0.30	1.0 (1)
**10. Have you ever felt like you would like to stop betting money or gambling, but you didn't think you could?**, yes, *n* (%)	4 (3)	152	2 (2)	2 (5)	0.65	0.2 (1)
**11. Have you ever hidden betting slips, lottery tickets, gambling money, I.O.U.s or other signs of betting or gambling from your spouse, children or other important people in your life?**, yes, *n* (%)	7 (5)	152	4 (4)	3 (7)	0.62	0.2 (1)
**12. Have you ever argued with people you live with over how you handle money?**, yes, *n* (%)	19 (13)	152	14 (13)	5 (12)	0.89	0.2 (1)
**13. (If you answered "yes" to question #12) Have money arguments ever centred on your gambling?**, yes, *n* (%)	2 (1)	19	2 (2)	0 (0)	0.96	0.0 (1)
**14. Have you ever borrowed from someone and not paid them back as a result of your gambling?**, yes, *n* (%)	1 (1)	152	0 (0)	1 (2)	0.62	0.3 (1)
**15. Have you ever lost time from work (or school) due to betting or gambling?**, yes, *n* (%)	6 (4)	152	1 (1)	5 (12)	0.008	7.0 (1)

**16. If you borrowed money to gamble or to pay gambling debts, whom/where did you borrow?**						
A. from household money, yes, *n* (%)	7 (5)	152	105 (95)	40 (95)	0.71	0.1 (1)
B. from your spouse, yes, *n* (%)	0 (0)	152	0 (0)	0 (0)	1	0.0 (1)
C. from other relatives or in-law, yes, *n* (%)s	2 (1)	152	2 (2)	0 (0)	0.93	0.0 (1)
D. from banks, loan companies, or credit unions, yes, *n* (%)	1 (1)	152	1 (1)	0 (0)	0.62	0.2 (1)
E. from credit cards, yes, *n* (%)	1 (1)	152	1 (1)	0 (0)	0.62	0.2 (1)
F. from loan sharks, yes, *n* (%)	1 (1)	152	1 (1)	0 (0)	0.62	0.2 (1)
G. cashed in stocks, bonds or other securities, yes, *n* (%)	1 (1)	152	1 (1)	0 (0)	0.62	0.0 (1)
H. sold personal or family property. yes, *n* (%)	2 (1)	152	2 (2)	0 (0)	0.93	0.2 (1)
I. borrowed on your checking account, yes, *n* (%)	0 (0)	152	0 (0)	0 (0)	1	0.0 (1)
J. have (had) a credit line with a bookie, yes, *n* (%)	0 (0)	152	0 (0)	0 (0)	1	0.0 (1)
K. have (had) a credit line with a casino, yes, *n* (%)	0 (0)	152	0 (0)	0 (0)	1	0.0 (1)
